# Language, agency, and deliberation: a multilevel theoretical framework for the computational analysis of citizen participation

**DOI:** 10.3389/frai.2026.1878502

**Published:** 2026-10-21

**Authors:** Claudio Fuentes-Bravo, María Paz Raveau

**Affiliations:** 1 Faro, Universidad del Desarrollo, Santiago, Chile; 2 Facultad de Derecho, Universidad de Chile, Santiago, Chile

**Keywords:** citizen participation, computational psycholinguistics, deliberative theory, discourse analysis, language style matching, LLMs, narrative identity, semantic roles

## Abstract

Computational analysis of citizen participation discourse has developed a substantial toolkit without a corresponding account of which linguistic dimensions carry psychological and political information. This article proposes a multilevel framework to fill that gap and states its claims as hypotheses rather than findings. Three analytical dimensions are identified: content, which covers lexical and topical features of what citizens say; interactional synchrony, which covers Language Style Matching (LSM) and the coordination of function words between participants; and narrative positioning, which aggregates semantic role profiles across a corpus to characterize the stance from which citizens narrate their relation to institutions. These dimensions are treated as complementary rather than as an ordered progression. We derive the framework from a decade of work with Chilean participatory corpora and place it in dialogue with deliberative theory, arguing that low interactional synchrony counts as evidence against communicatively oriented exchange while high synchrony does not count as evidence for it. This asymmetry turns the framework into an instrument for detecting deliberative failure.

## 1 Introduction

Digital platforms for citizen participation now generate textual corpora at a scale that exceeds human reading capacity. Thousands of proposals, comments and arguments accumulate within weeks, and the demand for computational treatment follows directly (Romberg and Escher, 2024; Arana-Catania et al., 2021). The tooling has responded with topic detection, sentiment classification, proposal identification and measures of citizen competence (Guridi et al., 2025). These methods have real operational value and will improve as models improve.

Their growing technical power leaves one problem untouched. Before asking how to analyze citizen language, we need an account of which dimensions of discourse are psychologically and politically informative, and why. Consider two contributions to a municipal consultation. One reads "the State should consider our point of view"; the other reads "we demand that the State fulfil its commitment." Topic modeling assigns them to the same cluster and sentiment analysis to the same valence. But they encode different relations to institutional authority. The difference is not hidden from the tools by any technical limitation; the questions guiding the analysis simply do not point at it.

Large language models sharpen the problem. Fluent replication of the surface of citizen discourse is now cheap (Brown et al., 2020), and human judges fail to distinguish model-generated from human contributions under controlled conditions (Jones and Bergen, 2025). Whether lexical indicators that once served as proxies for authentic participation still discriminate has not been tested on participatory material, and we expect that they do not. The response cannot come from better classifiers alone.

What we propose here is a framework for asking better questions. It offers no single implementation pipeline. It assembles analytical categories drawn from psycholinguistics, computational linguistics and semantic role analysis, and specifies which dimensions of language are informative about the political experience of citizens. Technical development makes the processing of any textual dimension more efficient; it does not adjudicate which dimensions deserve processing. That adjudication is theoretical work, and this article aims to carry it out.

The framework is organized along four axes. The first is explanatory: it moves from correlations between lexical patterns and psychological states toward the mechanisms that generate them. The second is interactional: it treats function words and their synchronization between speakers as evidence about the relation participants build with one another. The third is narrative: it aggregates semantic roles across a corpus to characterize the position from which each actor speaks. The fourth is diagnostic: it specifies an asymmetric inference under which the framework detects deliberative failure without claiming to certify deliberative success.

Two lines of recent work provide the immediate background. *Relatio* (Ash et al., 2024) demonstrates that narrative structures can be extracted from political corpora through semantic role labeling, and *Riveter* (Antoniak et al., 2023) operationalizes power and agency through verbal connotation frames. Structured prompting of LLMs has produced promising results in classification and deliberative assessment (Nie et al., 2025). All three operate on the syntagmatic axis, mapping role distributions within sentences or short sequences. Riveter does aggregate across a collection, returning an average power or agency score for each entity in the corpus, and that capability is closer to what we propose than the sentence-level tools are. What it returns is a scalar per entity. Our proposal requires the full distribution of roles an actor occupies, the entities it co-occurs with in high- and low-power positions, and the movement of both across the process, projected onto an explicit structural description of narrative position. Supplying that projection and specifying the annotation scheme and agreement statistic under which it can be carried out, is the contribution we defend.

The reasoning that produced the framework is abductive in Peirce's sense: a surprising observation resisted explanation by available theories and prompted the formulation of a hypothesis to render it intelligible (Peirce, 1934). Brandt and Timmermans (2021) argue that abduction suits computational social science, where digital data routinely yield findings that existing theory did not anticipate. Abduction generates hypotheses. It does not establish them, and the sections that follow are written with that limitation in view.

### 1.1 Epistemic status of the claims

A framework that proposes analytical categories in advance of their validation carries a specific obligation, which is to state what it takes to prove it. Abductive inference does not establish construct validity, measurement properties in a given corpus, or practical utility for institutional evaluation. We therefore classify the central claims of this article into three categories.

Established (E) claims are supported by convergent published evidence. Transferred (T) claims rest on findings obtained in other domains; their extension to Spanish-language citizen deliberation is an assumption the framework makes and does not verify. Hypothetical (H) claims originate here, lack direct support, and are stated together with the observation that would refute them.

The boundary between E and T marks the largest silent assumption in this line of work. Most psycholinguistic evidence underpinning the first two levels was obtained in English, in dyads or small groups, among participants who addressed no institution and whose contributions carried no expectation of policy consequence. Deliberative citizen participation departs from all four conditions. This holds for constituent processes as much as for participatory budgeting or municipal consultations, though the departure is sharpest where policy consequence is most salient, as in constitutional settings. Treating that transfer as unproblematic would repeat the error we attribute to lexical approaches, letting the availability of a measure determine what gets measured.

Two consequences follow. Claims about what the framework reveals should be read as claims about what it would reveal if the hypotheses in Table 1 hold. And the framework admits a global falsifier. On a corpus where topic, sentiment, semantic role distribution and structural position are jointly measured, if the role and position variables account for no interpretive or predictive variance beyond that captured by topic and sentiment, the central premise of this article fails and the lexical paradigm we criticize is adequate as it stands. Designing that comparison is the first task of the empirical programme the framework opens.

**Table 1:** Epistemic status of the article's central claims.

#	Claim	Status	Basis, or condition that would refute it
	Content dimension		
1	Content-word frequencies index attentional focus and covary with psychological states	E	Tausczik and Pennebaker (2010); Pennebaker and King (1999); Yarkoni (2010); Schwartz et al. (2013)
2	Dictionary-based lexical counting is insensitive to the positional and relational structure of an utterance	E	Follows from the construction of the measures
3	Lexical markers do not discriminate authentic contributions from LLM-generated ones	T	Jones and Bergen (2025) show that human judges fail to discriminate in controlled interaction; the extension to lexical measures applied to written participatory contributions is an assumption. Refuted if dictionary-based measures separate human from model-generated contributions in a matched participatory corpus.
	Interactional synchrony		
4	Function words are produced under less deliberate control than content words	E	Chung and Pennebaker (2011)
5	LSM indexes interactional engagement between interlocutors	E	Niederhoffer and Pennebaker (2002); Gonzales et al. (2010); Ireland et al. (2011)
6	LSM is valence-neutral; high LSM occurs in conflict, negotiation and impasse	E	Ireland and Henderson (2014); Taylor and Thomas (2008)
7	LSM transfers to Spanish deliberative corpora using Spanish function-word categories	T	Refuted if Spanish categories fail to reproduce the reliability and dyadic sensitivity reported for English
8	Low citizen–institution LSM is evidence against communicatively oriented interaction	H	Refuted by processes combining low LSM with high independently coded uptake and documented position change
9	Under favorable deliberative conditions, interindividual synchrony exceeds intergroup synchrony once both are expressed relative to level-specific null distributions	H	Refuted where the ordering fails to appear, or appears independently of any process-quality indicator
	Narrative positioning		
10	Semantic role profiles can be extracted and aggregated per actor across a deliberative corpus	T	Feasibility shown for English political corpora (Ash et al., 2024; Antoniak et al., 2023); unverified for colloquial deliberative Spanish
11	Structural positions can be assigned to aggregated profiles with acceptable inter-annotator agreement	H	Refuted by agreement at or below chance in a two-annotator trial
12	Agent-dominant narration covaries with subjective political efficacy	H	Refuted by absence of association between role profiles and efficacy scores co-measured in the same participants
13	Narrated agency dissociates from objective institutional influence, and the dissociation widens under designs that grant voice without decision power	H	Refuted if narrated agency tracks documented incorporation of citizen input into institutional outputs

The remainder of the article presents the trajectory that motivated the framework, develops the three levels, places them in dialogue with deliberative theory, describes implementation stages, and states the scope conditions under which the proposal should be read.

## 2 A decade with Chilean participatory corpora

Between 2015 and 2024 our team worked on corpora from a series of Chilean participatory processes. Each project imposed its own constraints: the material the convening organization could supply, the questions it needed answered, and the resources available at the time. The framework proposed here reconstructs, after the fact, what that sequence of ad hoc decisions kept running into. That work covered the citizen phase of the constituent process under the Bachelet government (Consejo Ciudadano de Observadores, 2017), the popular participation instruments of the Constitutional Convention (Fuentes et al., 2022), the town halls of the "Tenemos que Hablar de Chile" project in its successive versions (Tenemos que Hablar de Chile, 2022, 2024), and a comparative review of 54 citizen dialogue initiatives conducted for the Executive Secretariat of Citizen Participation (Secretaría Ejecutiva de Participación Ciudadana, 2023).

Two limitations recurred across these projects, and Table 2 summarizes them alongside the field-level developments that were occurring in parallel.

**Table 2:** Recurring limitations across two methodological regimes.

Aspect	Specialized NLP (2015–2021)	Generative models (2022–2024)
Tools	Lexical counting, topic modelling, sentiment classification, mostly trained on English	Structured prompting, transformer-based SRL, narrative extraction tools such as *Relatio* and *Riveter*
What worked	Thematic summaries at scale; frequency profiles by sociodemographic segment; emotion classification	Richer interpretive distinctions, including position of enunciation, from colloquial and regionally marked Spanish
What did not	Thematic results did not translate into institutional decisions. Knowing that "fairness," "health" and "education" are the most frequent concepts does not indicate how to incorporate citizen voice into a constitutional text	Replicability and auditability degraded. Manual annotation across processes exposed disagreement over categories such as "demand," "proposal" and "complaint," which had no shared definition
Question left open	Which structural dimensions of citizen discourse can be measured, given that lexical and thematic analysis leaves the position of enunciation invisible?	Which analytical categories are precise enough to guide annotation, rich enough to capture political stance, and stable enough to permit comparison across heterogeneous designs?

The first limitation was practical. Frequency-based results described what concerned citizens without indicating from what political position the concern was voiced, and institutional users found them difficult to act on. The second was methodological. When we attempted comparison across the 54 processes, the absence of shared analytical categories made systematic comparison impossible, and manual annotation exposed conceptual ambiguities that better tools would not have resolved.

These are local observations, and we do not present them as evidence. They function here as the anomaly that motivated an abductive move: the shortfall was conceptual, and no optimization of the existing measures addressed it. The lexical-frequency paradigm rests on the assumption that content words are the primary bearers of psychological and political meaning in a text, and it was that assumption, rather than the accuracy of any particular implementation, that our results kept encountering. Branahl (2025) offers a useful characterization of research programmes that continue to accumulate results while ceasing to generate questions their core did not already contain. Whether the framework proposed here constitutes a progressive alternative in Lakatos's sense (Lakatos, 1978) is a judgment that can only be made retrospectively, once the hypotheses in Table 1 have been tested.

## 3 Three complementary perspectives

The framework distinguishes three analytical dimensions, each answering a different question about the same corpus. Content analysis asks what the citizen says about the world, which topics they raise, which actors they name, which emotions they express. The citizen is an object of observation. Interactional synchrony shifts to the relation between participants, asking how two interlocutors affect one another in exchange and whether their styles converge. Narrative positioning asks from what position the citizen speaks, what role they occupy in the account they give, and whether that position persists across the process. Nothing in this distinction implies that one dimension is more fundamental than another; each targets a different aspect of the text and each has its own conditions of applicability.

### 3.1 Content: lexicon, attention, and its limits

The first level corresponds to the paradigm that has dominated computational psycholinguistics since the late 1990s: content-word frequencies as proxies for psychological states. The researcher observes what the citizen says without asking how the utterance is relationally structured or from what position it is issued. The founding principle, developed by Pennebaker and colleagues, holds that lexical frequencies reflect foci of attention and, through attention, underlying psychological states (Tausczik and Pennebaker, 2010).

The approach has demonstrated descriptive and predictive power in its domain of origin. Supported by LIWC (Linguistic Inquiry and Word Count) and comparable instruments, it has mapped lexical patterns onto personality traits, physical and mental health, and a range of social behaviors (Pennebaker and King, 1999; Yarkoni, 2010; Schwartz et al., 2013). Its appeal for large participatory corpora is easy to understand. It is operationally simple, computationally cheap, and yields results comparable across corpora of any size. It was the entry tool for the first Chilean corpora we worked with, and it remains the appropriate first pass.

Its limitation was stated precisely by Boyd and Schwartz (2021), following Meehl's (1990) critique of intercorrelation networks that catalog associations without specifying mechanisms. The paradigm treats content words as direct carriers of psychological meaning and leaves the mediating processes unexamined. Applied to citizen participation, this produces a specific blind spot. A citizen who writes "we have gone years without a response from the municipality" and one who writes "we demand that the municipality respond once and for all" share vocabulary, topic and emotional valence while occupying different positions with respect to institutional authority. Lexical analysis does not register the difference.

A second fragility follows from the first. Any system that replicates the vocabulary of citizen participation would produce text that lexical measures cannot separate from genuine contributions, and current models meet the generative condition (Brown et al., 2020), while the evidence that discrimination fails comes so far from human judges rather than from dictionary-based measures applied to participatory corpora (Jones and Bergen, 2025). The gap is recorded as claim 3 of Table 1. This is not an argument for abandoning the level. It is an argument for treating it as necessary and insufficient.

### 3.2 Interactional synchrony: function words and coordination dynamics

The second level shifts the unit of analysis from the individual to the dyad. The question is no longer what a participant says but how two participants affect one another over the course of an exchange, and what their linguistic production reveals about the attention each pays to the other.

The shift rests on a distinction with technical consequences. Content words are deliberate selections that a speaker can control strategically. Function words are processed faster, more automatically and with less conscious access (Chung and Pennebaker, 2011). Their distribution is a stylistic habit that largely escapes intentional management, which makes them informative about interactional dynamics in a way that content words are not. Language Style Matching (LSM) operates on this vocabulary, measuring convergence in function-word use between two participants in conversation (Gonzales et al., 2010; Niederhoffer and Pennebaker, 2002). Neither speakers nor trained observers detect the phenomenon reliably (Ireland and Pennebaker, 2010). Its predictive value has been demonstrated in speed-dating studies, where higher LSM accompanied stronger mutual romantic interest, and in established relationships, where it predicted stability over time (Ireland et al., 2011).

Conversational Semantic Role Labeling (CSRL) extends this perspective. Where standard Semantic Role Labelling (SRL) operates within sentences, CSRL identifies predicate arguments distributed across conversational turns, recognizing that in dialogue the semantic structure of an utterance is often completed by the interlocutor (Fei et al., 2022; Xu et al., 2021). LSM measures the degree of interactional engagement; CSRL maps the argumentative content that engagement carries.

#### 3.2.1 What LSM licenses, and what it does not

The evidence base for LSM comes substantially from strategic settings. Taylor and Thomas (2008) studied negotiation, where convergence accompanied successful resolution. Ireland and Henderson (2014) found that in conflict contexts synchrony can amplify hostility and predict impasse. Neither result supports reading high LSM as a marker of communicatively oriented exchange. Accommodation is compatible with persuasion, impression management, institutional scripting, shared topic, demographic similarity and automatic mimicry, and it has been shown that language models accommodate syntactically to their conversational partner as well (Kandra et al., 2025).

We therefore propose an asymmetric reading. Low synchrony between citizens and institutional respondents counts as evidence against communicatively oriented interaction, since mutual attention is a necessary condition of exchange oriented toward understanding. High synchrony does not count as evidence for it, since the same pattern arises from strategic accommodation. The asymmetry is not a weakness of the measure. It positions the framework as an instrument for detecting deliberative failure, which for institutional evaluation is the more useful capability: a process can be certified as having failed to produce mutual attention on evidence that a positive certification would not support.

This reading has a direct implication for how results should be reported. A study using LSM to characterize a participatory process should state which pattern would have counted against its conclusion, and should refrain from converting high synchrony into a quality score.

#### 3.2.2 Confounders and null models

Three sources of artefact require explicit treatment before any LSM value can be interpreted.

The first is topic. Function-word distributions covary with subject matter, since legal, budgetary and territorial discussions impose different distributions of prepositions, articles and quantifiers. Any comparison must either hold topic constant or model it. Corpora annotated with a policy-topic scheme make this tractable without additional coding.

The second is text length. Function-word proportions are unstable in short texts, and citizen contributions are frequently short. Studies should report a minimum token threshold, match compared units on length and include a sensitivity analysis identifying the length at which the measure stabilizes in the corpus.

The third is aggregation. When individual texts are pooled into a group profile, sampling variance in the underlying proportions is reduced, and two aggregated profiles will tend to resemble one another more than two individuals drawn from the same population. A naive comparison of group-level and individual-level synchrony will therefore report higher values at the group level for arithmetic reasons alone. The remedy is a level-specific null model: participants are randomly reassigned to pseudo-groups, the statistic is recomputed across many reassignments, and the observed value is reported as a deviation from the resulting distribution. Values from different levels should never be compared in raw form.

Beyond artefact control, the measure requires validation against independent evidence. Three candidates are available at reasonable cost: turn-by-turn coding of uptake by two annotators on a subsample, with agreement reported; documented position change between successive rounds of the same process; and the subscales of the Discourse Quality Index applied to the same material.

A separate assumption concerns language. Function-word categories developed for English do not transfer isomorphically to Spanish, which licenses null subjects, encodes person in verbal inflection and uses clitics where English uses free pronouns. Personal pronoun frequency, one of the most informative categories in the English literature, measures something different in Spanish, where its presence often marks contrast or emphasis rather than reference. Any application of LSM to Spanish deliberative material should compare at least two category schemes and report the sensitivity of results to that choice. We propose a concrete form for that comparison. One scheme can be built from the Spanish adaptation of LIWC (Ramírez-Esparza et al., 2007), which remains available as a built-in dictionary in current LIWC releases. That adaptation translates an English dictionary rather than rebuilding its categories around Spanish morphosyntax, so it cannot by itself settle the question raised above, and the second scheme should, consequently, derive its categories from morphological notation, treating clitics and null subjects as classes in themselves. Claim 7 of Table 1 should then be treated as holding only where both schemes reach a split-half reliability of at least .70 across the resulting scales and agree in the direction of the dyadic differences they report. We offer that threshold as a proposal rather than as an established convention, since the LSM literature has not standardized a reliability criterion.

Because comparability with the psycholinguistic literature and auditability are central to the framework's purpose, the classical pipeline is the primary implementation route. The prompted alternative is reserved for corpora whose conversational structure does not yield identifiable turn pairs.

#### 3.2.3 A multilevel architecture for LSM in deliberative settings

Synchrony in a deliberative process cannot be captured by a single measurement. Interaction among citizens, dynamics within and between groups, and the relation between citizens and the convening institution raise distinct questions and require distinct comparisons. The architecture below organizes them into three levels whose cross-comparison constitutes the framework's principal diagnostic instrument. Throughout, values are understood as deviations from the level-specific null described in Section 3.2.2.

*Interaction among citizens*. The starting point is synchrony between individual participants. This level offers the closest approximation to spontaneous engagement, because citizens do not operate under the professional register constraints that shape institutional speech, and their style remains sensitive to the interlocutor. Where turns are individually identifiable, the level can be extended through CSRL, which reconstructs how participants distribute the semantic structure of an exchange across turns (Xu et al., 2021).

*Dynamics among groups*. The second level admits two segmentations. The first operates on the deliberative groups constituted by the process itself, permitting a comparison between synchrony within groups and synchrony between them. A large gap indicates that deliberation produced local stylistic communities rather than a shared communicative space, and that the minipublic functioned as an aggregate of interactional enclaves. Note that intergroup and interindividual synchrony measure different things: the former compares aggregated stylistic profiles, describing the state each group reached after its own internal deliberation, while the latter captures adaptation between interlocutors as a process. The null models make the two comparable in interpretation, not in kind.

The second segmentation partitions participants by criteria external to the process, such as educational attainment, age or area of residence. Synchrony computed over these segments does not measure an effect of deliberation. It estimates the stylistic distances participants brought with them, and functions as a sociological baseline against which differences observed at other levels can be assessed.

*Citizen–institution relation*. The third level applies the asymmetric reading from Section 3.2.1 to exchanges between citizens and the institutional respondent. This could be a citizen dialog and the body issuing a consolidated response, or an individual submission and its official reply. Low synchrony here counts as evidence that the institution answered from its own categories without registering what the citizen said. High synchrony does not certify the opposite, since institutional accommodation can also come from a scripted or strategic response. Hypothesis 8 of Table 1 is tested at this level. Throughout this level, “low” and “high” refer to deviations from the null distribution described in Section 3.2.2 rather than to a fixed value, since citizen and institutional registers differ structurally in ways that would otherwise be mistaken for a synchrony effect. The normative stakes are clearest here too: a participatory design can be shown to have failed at producing mutual attention, but no reading of this measure certifies that it succeeded.

*Cross-level comparison as diagnosis*. The contribution of the architecture lies in the patterns that emerge from comparing levels rather than in any single value. Where citizens synchronize with one another but groups do not converge, deliberation produced local cohesion without integration. Where synchrony between demographic segments is low while synchrony between individuals is high, the process attenuated communicative distances that operate structurally. Where synchrony within sessions is high but synchrony across sessions is low, coherence was local rather than cumulative. Single-level analysis distinguishes none of these. Where synchrony among citizens is high but citizen–institution synchrony is low, deliberation succeeded internally without reaching the convening body. Without the citizen-level comparison as a baseline, this pattern is indistinguishable from institutional failure.

We expect synchrony among individual citizens to exceed intergroup synchrony once both are expressed relative to their nulls, with greater variability between groups than within them. Systematic violation of this ordering would be a substantive finding about process design, group composition or the citizen–institution relation, and it is stated here as hypothesis 9 of Table 1 precisely so that it can fail.

### 3.3 Narrative positioning: semantic roles and narrated agency

The third level asks from what position the citizen speaks, what role they occupy in the account they give of the process, and whether that position persists over time.

The position is accessible through semantic role labeling (SRL), which identifies the function each constituent performs in the situation described by the verb: who acts deliberately, who undergoes the action and changes state, where the action is directed, where it originates (Palmer et al., 2005, 2010). Applied to isolated sentences, SRL yields a local description. Applied across a full corpus of interventions and aggregated by actor, it yields a profile: the relative frequency with which an entity appears as agent or as patient, the power valence of the verbs that take it as argument, and the evolution of both across the process.

#### 3.3.1 Four senses of agency

The relation between such a profile and the political standing of the person who produced it requires care, because four distinct properties are easily conflated.

1. Grammatical agency. Occupancy of the agent role in a semantic role structure. A property of the text.
2. Narrated agency. The position an actor occupies in the account given across the corpus, characterized by the aggregated profile and its structural projection. A property of the discourse.
3. Subjective political efficacy. The belief that one's action can influence collective outcomes. A psychological property, measurable by validated scales.
4. Objective institutional influence. The traceability of a participant's input into institutional output. A political property, measurable from documentary evidence.

The framework measures the first two. Their relation to the third and fourth is an open question, and the reasons for caution are substantive rather than merely methodological. Demand language often indexes lack of access, since what can be obtained directly need not be demanded. Actors with institutional power frequently write in nominalizations and agentless constructions that suppress grammatical agency altogether. In settings of pronounced asymmetry, which describes the Chilean processes we have worked with, the mapping from grammatical to subjective agency may run in the opposite direction from the intuitive one. This caution has a normative dimension as well as a methodological one. A Patient-dominant profile can register an accurate account of institutional neglect rather than a psychological deficit. Treating it as something to be corrected would repeat the error Sanders (1997) and Young (2000) attribute to deliberative standards built on argumentation alone, where testimony about exclusion gets read as a communicative failure of the speaker rather than as evidence about the process. The framework tracks position. It does not assign merit to one position over another.

Stating the four senses separately makes a testable prediction available. Under conditions of institutional capture, where a participatory design grants voice without transferring decision power, narrated agency should rise while objective influence remains flat. That dissociation would be detectable in the linguistic record before it manifested as withdrawal from participation, and the lexical paradigm cannot anticipate it. It appears as hypothesis 13 of Table 1.

#### 3.3.2 From local roles to corpus-level position

Recent work has extended role analysis beyond the sentence. CSRL recovers predicate arguments distributed across turns (Fei et al., 2022). *Relatio* extracts narrative structures from role sequences in political corpora (Ash et al., 2024). The power and agency connotation frames of Sap et al. (2017), implemented in *Riveter* (Antoniak et al., 2023), add a layer describing the power relations implicit in verb choice.

These tools share a scope. *Relatio* and CSRL describe role distribution locally, within sentences or short sequences, and *Riveter*, while it does aggregate across a corpus, reduces each actor to an average connotation score. What none of them delivers is a characterization of the position an actor occupies as a structure rather than as a magnitude: not how much power a citizen is attributed on average, but what a citizen consistently does and undergoes over the course of an entire participatory process, in relation to which other actors, and how that configuration can be assigned to a structural position under a scheme that another analyst could apply to the same material and reach comparable results. Supplying that projection is the contribution we defend, and it is separable from any particular vocabulary for describing the result.

For Spanish, the AnCora corpora (Taulé et al., 2008) are annotated for semantic role analysis, and its extension, AnCora-Co (Recasens and Martí, 2010), is enriched with coreference information. Both sources consist mainly of newspaper text, so they set an upper bound on what can be achieved elsewhere. It does not establish validity for colloquial deliberative Spanish, and the verification hypothesis 10 calls for therefore requires an annotated subsample of the participatory corpus itself, with the agreement statistic reported on that subsample rather than on the reference corpora.

Three projections of an aggregated profile onto a structural description are available, and they differ in the interpretive burden they impose.

The most conservative extends the connotation-frame approach already validated at sentence level. An actor is characterized by the distribution of power and agency valences attributed to it across the corpus, together with the entities with which it co-occurs in high- and low-power positions. The categories are those of Sap et al. (2017), the aggregation is new, and the annotation task inherits an existing definition.

A second projection uses force-dynamic schemas (Talmy, 2000), which describe configurations of an entity with a tendency toward change and an entity that blocks or yields. The mapping onto the citizen–institution relation is direct, the categories are few and binary, and the framework has current standing in cognitive linguistics.

A third uses positioning theory (Davies and Harré, 1990), which has an established coding tradition in political discourse analysis and addresses speaking rights and identity claims explicitly.

Structuralist narrative models, and in particular the actantial model (Greimas, 1983, 1987; Wang and Roberts, 2005), constitute a fourth possibility. However, the model presupposes a quest structure, a subject pursuing an object, which fits extended narrative but not the proposals, demands and short complaints that make up much participatory material; where the structure is absent, the analyst supplies it. And its categories admit multiple defensible assignments over the same text, which is acceptable in literary semiotics and problematic in a framework offering comparability across processes. We include it as one available projection rather than as the framework's core.

Whichever projection is adopted, the requirement is the same: an explicit annotation scheme, a reported agreement statistic between independent coders, and a stated procedure for resolving disagreement. Hypothesis 11 of Table 1 states that this is achievable and specifies what would show that it is not.

## 4 The framework and deliberative theory

The limitations our team encountered when analyzing participatory corpora correspond to problems that normative theory identified decades ago and could not measure at scale. This section sets out the correspondence, identifies what remains unresolved, and situates the framework with respect to two literatures whose absence from computational work on deliberation is conspicuous.

### 4.1 Communicative action and the asymmetric reading

The relevant starting point is Habermas's distinction between strategic and communicative action. Strategic action describes interaction in which participants seek to move others toward predetermined ends; communicative action describes interaction in which participants seek mutual understanding and remain open to revising their position in the light of better argument (Habermas, 1984). Democratic legitimacy, on this account, depends on the transformation of preferences through exchange among equals rather than on the aggregation of preferences formed in advance (Habermas, 1996).

The distinction has resisted computational operationalization. Lexical methods identify what participants say and leave open whether they are attending to one another's arguments or reproducing fixed positions. The second level of the framework addresses this, though under the constraint developed in Section 3.2.1. A citizen–institution exchange marked by low synchrony provides evidence that the institution is responding from its own categories without the citizen's contribution having registered. The converse inference is unavailable. This is a narrower claim than the one normative theory would like to have, and it is the claim the evidence supports.

The content dimension speaks to a different aspect. Whether participants invoke common or particular interests, whether justifications are explicit, whether arguments are internally coherent: these are the dimensions the Discourse Quality Index (DQI) captures through manual coding (Steenbergen et al., 2003; Steiner et al., 2005). The DQI is grounded in discourse ethics and focuses on observable behavior, and it is confined by its coding requirements to parliamentary debates and minipublics. Lexical methods scale these dimensions to large corpora at a cost in interpretive depth.

The third level engages a question Habermas's framework treats as presupposed: who can speak, in what terms, and from what position. Participants in the ideal case regard one another as equals and assume that anyone may take part (Habermas, 1996). Real processes rarely satisfy this, and the empirical problem is detecting the shortfall. Aggregated role profiles offer one route. Where participants from particular social groups are systematically narrated as undergoing institutional action while others are narrated as performing it, the asymmetry is a linguistic trace of conditions that normative theory requires, and practice has not delivered. The four-way distinction of Section 3.3.1 restricts what may be concluded from such a finding: it describes the discursive record, and its relation to efficacy and influence remains to be established.

### 4.2 The narrative turn and its computational treatment

Narrative entered deliberative theory as a corrective. Sanders (1997) argued that a deliberative standard built on rational argumentation reproduces the exclusions deliberation is meant to overcome, and Young (2000) developed the case for greeting, rhetoric and narrative as legitimate modes of political communication. Updated versions of the DQI have responded by incorporating storytelling (Bächtiger et al., 2022).

That incorporation remains binary. Coders record whether a participant used personal narrative, which registers presence and leaves structure unexamined. The third level of the framework goes further, characterizing the position the speaker occupies with respect to other actors and the trajectory of that position across the process. We are not aware of a systematic computational development of the narrative turn along these lines, which is what makes the proposal worth stating and also what makes it untested.

McAdams's (1995) work on narrative identity suggests a normatively significant extension. Deliberation that changes preferences may also change the position from which participants narrate their relation to institutions. A participant who enters a process narrating themselves as undergoing institutional decisions and leaves it narrating themselves as acting collectively has undergone a change that preference-based measures do not capture (Adler et al., 2016, 2017). Whether such shifts occur, and whether they track anything beyond the discursive record, are open questions and appear in Table 1 accordingly.

### 4.3 The DQI, argument mining, and what the framework adds

Automated approximations to the DQI have been developed through machine learning (Fournier-Tombs and Di Marzo Serugendo, 2020). They inherit the instrument's scope, measuring the formal quality of argument without capturing the relational and positional dimensions that Sanders and Young identified as constitutive. A recognized limitation of the original index is that it captures formal properties of arguments and neither their substantive quality nor the sincerity with which they are offered (Steenbergen et al., 2003).

Argument mining supplies capabilities the framework does not duplicate and should not ignore. The field has developed methods for identifying claims and premises, reconstructing argumentative relations, and assessing argument quality in dialogic settings (Lawrence and Reed, 2019; Stede and Schneider, 2018). Where the DQI asks whether an argument meets a normative standard and argument mining asks how arguments are structured and related, the framework asks whether participants are attending to one another, from what position they issue their claims, and whether that position moves. The three are complementary, and a full account of deliberative quality in large corpora will require all of them. A specific case for the combination arises where a process satisfies formal criteria while low synchrony indicates that no participant is changing in response to another and role profiles show citizens occupying the same position at the end as at the beginning (Bächtiger and Parkinson, 2019).

### 4.4 Systemic scope and unresolved tensions

The deliberative systems literature has shifted the unit of normative assessment from the individual forum to the ecology of sites through which an issue moves (Mansbridge et al., 2012; Bächtiger et al., 2018). This bears directly on the framework's scope. Measurements taken within a single minipublic characterize that minipublic, and a process that fails on the framework's indicators may still contribute to a deliberative system through other functions, such as agenda formation or the transmission of claims into representative institutions. Diagnoses issued at forum level should not be read as system-level verdicts.

Three tensions remain. The first is between universality and context. Habermasian theory posits universal conditions of rational communication; the framework operates on particular corpora in particular political settings, and a given synchrony value in Santiago need not carry the meaning it carries in Oslo. Developing culturally calibrated interpretive criteria, and specifically Latin American ones, remains outstanding.

The second is between procedural and substantive quality, which the four-way distinction of Section 3.3.1 renders explicit rather than resolves. Establishing whether narrated agency corresponds to influence requires evidence external to the corpus.

The third is between description and intervention. Sætra (2024) argues that using AI to improve deliberation risks reducing it to language optimization and neglecting the engagement with conflicting values that gives deliberation its point. The objection applies to this framework when its indicators are used to design or moderate rather than to diagnose. We offer it as a diagnostic instrument, and its use as an intervention tool would require normative argument this article does not provide.

## 5 Implementation

The three perspectives capture complementary dimensions. Their integration in the analysis of actual corpora proceeds through stages that admit more than one implementation. The four stages developed below form a single sequence: corpus segmentation into narrative episodes (5.1); semantic role extraction within each episode, followed by coreference resolution and aggregation by entity (5.2); projection of the aggregated profile onto a structural description (5.3); and a choice between classical, prompted or hybrid implementations at each stage (5.4). The sequence runs from the sentence to the corpus, and each stage can draw on the classical or the prompted pathway summarized in Tables 3–5 independently of the choice made at the stage before it.

**Table 3:** Implementation of the content dimension.

Implementation feature	Classical pipeline	LLM-based pathway
Approach	Dictionary-based lexical counting	Prompted classification of excerpts into researcher-defined thematic and affective categories
Advantage	Scalable; comparable across corpora and time points; low cost	Flexible category definition; handles colloquial and regionally marked language
Limitation	Dictionaries developed for English; predefined categories not always pertinent to Chilean political discourse	Results not directly comparable with prior psycholinguistic literature; variability between runs

**Table 4:** Implementation of the interactional synchrony dimension.

Implementation feature	Classical pipeline	LLM-based pathway
Approach	Deterministic computation over function-word categories defined by the psycholinguistic literature	Prompted analysis of stylistic alignment between paired utterances
Advantage	Directly comparable with prior studies; replicable and auditable; low cost	Handles non-linear conversational structures common on digital platforms
Limitation	Requires exchanges delimited as turn pairs; limited resources for Spanish	Not comparable with prior literature; variability between runs; difficult to validate systematically
Recommended use	Corpora with deliberative turns segmented by process design	Corpora where turn pairing cannot be inferred from the design

**Table 5:** Implementation of the narrative positioning dimension.

Implementation feature	Classical pipeline	LLM-based pathway
Approach	Transformer-based SRL with coreference resolution and connotation lexicons	Prompted role labelling, reference resolution and power-connotation analysis in an integrated operation
Advantage	Deterministic and auditable; replicable across corpora and time points; each assignment justifiable by explicit criteria	Handles colloquial Spanish; reasons over extended context; requires no Spanish-specific resources
Limitation	Models trained largely on English and formal registers; degraded performance on colloquial deliberative Spanish	Assignment criteria not auditable; variability between runs; cost at scale

### 5.1 Segmenting the corpus

Citizen deliberative discourse has discursive properties that general-purpose models do not always handle: an argumentative structure oriented toward an institutional addressee, and a register that moves between colloquial and formal (Romberg and Escher, 2024). The unit of analysis we propose is the narrative episode, a sequence of statements describing an event with a beginning, a middle and an end.

The following passage serves as an illustration throughout this section:

*“For years we have demanded that our rights be recognized in the new Constitution. The Convention listened to us at first, but in the end the communities' proposals were left out of the text. As an organization we have had to keep pushing on our own so that our voice does not disappear from the debate.”*

Treated as a single episode, the passage exhibits a trajectory that its three sentences considered separately would not show. The organization appears first as a collective agent making a demand, then as undergoing an institutional process that excluded its proposals, and finally as recovering partial agency through autonomous action. Correct segmentation is a precondition for the later stages to register this. Classical implementations use syntactic parsers and clause boundary detection; prompted implementations request identification of episodes by argumentative structure.

### 5.2 Aggregation and profile construction

Roles extracted sentence by sentence do not by themselves characterize an actor's position across a corpus. This stage aggregates by entity, producing for each actor the relative distribution of roles it occupies, the power valence of the verbs that govern it, and the evolution of both across the process.

Coreference resolution is required, and in constituent corpora it is unusually difficult. Citizens refer to institutional actors through expressions that are ambiguous by design: "the Convention," "the constituents," "they," "the State," "those at the top." A participant who says "they" without specification may mean the Convention, the government, the political class or a diffuse abstraction of power, and the ambiguity is itself information about how democratic institutions are perceived. Classical implementations group mentions with coreference models; prompted implementations reason over context, including institutional and ambiguous reference.

### 5.3 Structural projection

This stage carries the framework's interpretive weight. Aggregated profiles are projected onto one of the structural descriptions set out in Section 3.3.2. The projection is an inference rather than a translation, and it should be conducted under an explicit annotation scheme with reported agreement.

Read through aggregated connotation frames, the illustrative corpus would characterize civic organizations by a profile combining high-agency verbs in the demand register with low-power positions in verbs governed by institutional actors, and the Convention by a profile in which listening co-occurs with exclusion. The interpretive content lies in the co-occurrence, and the absence of any actor that legitimates the citizen demand and guarantees its traceability into constituent decisions is itself a finding about the design of the process. The projection goes beyond the average score such a package returns, since the interpretive content lies in the co-occurrence structure rather than in the magnitude. Read through a force-dynamic schema, the same material yields a configuration in which an entity with a tendency toward change encounters an entity that yields and then blocks. Both descriptions are recoverable from the aggregated profile; neither is recoverable from sentence-level analysis.

Classical implementations translate profiles into assignments through explicit heuristics; prompted implementations require precise category definitions and worked examples.

### 5.4 Choosing between implementations

Neither route dominates. Replicability favors the classical pipeline, since comparable indicators across processes or time periods depend on deterministic behavior. Adaptability favors the prompted route, particularly for colloquial Spanish and for domains distant from the training material of classical tools. Auditability favors the classical pipeline wherever analysis informs policy design, and each decision must be explicable. Interpretive depth favors the prompted route at the projection stage.

For most research settings a hybrid is likely to be preferable: classical tools for extraction and aggregation, where replicability matters most, and prompted analysis at the projection stage, where interpretive flexibility contributes most. What the framework fixes is the question; the tools are chosen to answer it.

## 6 Scope conditions

Three limitations bound the proposal and are stated here rather than left to be discovered.

Authenticity. The proliferation of generative models raises a problem the framework does not solve. If synthetic contributions are indistinguishable from genuine ones in lexicon, syntax and topic, content-based methods lose diagnostic value for assessing the integrity of a participatory process, and the consequence is political rather than merely methodological. It is tempting to suppose that the deeper levels of the framework supply a remedy, on the grounds that longitudinal coherence and reciprocal accommodation require an interactional history that a model lacks. We do not think this survives examination. This closes off a broader claim as well: nothing established here supports treating narrative positioning as inherently harder to simulate than content, or interactional synchrony as an intermediate case between the two. The three dimensions differ in what they measure, not in how resistant they are to imitation. Models accommodate syntactically to their interlocutor (Kandra et al., 2025), pass controlled discrimination tests (Jones and Bergen, 2025), and produce outputs that converge with human outputs while arriving at them by different means (Loru et al., 2025; Quattrociocchi et al., 2025). Any observable property of linguistic production is in principle reproducible by a system with access to interaction history, a persistent persona and adequate instruction. Discriminative signatures of probabilistic generation do appear to exist, but at the level of statistical regularity in the text rather than at the level of the constructs proposed here.

A second consideration weighs against using these indicators as an authenticity filter even if they were shown to discriminate. The authentic participants most likely to display low synchrony are those being ignored, and those most likely to display unstable positional profiles are those whom the process has displaced. A filter built on these markers would penalize precisely the participants the narrative turn sought to include. Where such indicators are used at all, they belong in the diagnosis of processes, not in the screening of contributions.

Language and setting. The framework's second level rests on findings obtained in English and in settings without institutional addressees, and its transfer is an assumption rather than a result. The Spanish function-word question of Section 3.2.2 is a precondition for any application, not a refinement of one.

Level of analysis. The framework characterizes forums. Extending its indicators to the assessment of deliberative systems would require an account of how forum-level properties aggregate, which the systems literature has developed normatively, and no computational treatment has yet operationalized.

## 7 Conclusion

Computational analysis of citizen participation faces a difficulty that is theoretical before it is technical. Available tools process deliberative text with increasing efficiency and cannot determine which dimensions of that text carry psychological and political information. That determination requires a framework specifying why some linguistic structures matter more than others for understanding the political experience of citizens.

The framework proposed here rests on four claims whose validity is independent of any implementation. The first is explanatory: linking structural patterns in language to specific processes, rather than cataloguing correlations, makes linguistic analysis a route to understanding rather than to measurement alone. The second is interactional: function words and their synchronization carry information about the relation participants build with one another that content analysis does not reach. The third is positional: aggregating semantic roles across a corpus, and projecting the result onto an explicit structural description, characterizes the stance from which citizens address the state and other actors. The fourth is diagnostic: the asymmetric reading of synchrony allows a process to be identified as having failed to produce mutual attention on evidence that would not support the corresponding positive verdict.

Two features of the proposal deserve emphasis at the close. Its independence from any particular computational strategy means its relevance is not tied to a technology in a field changing at speed; the questions it poses can be pursued through classical pipelines, prompted architectures or hybrids. And its claims are stated as hypotheses with refutation conditions, including the global falsifier stated in Section 1.1. On a corpus where topic, sentiment, semantic role distribution and structural position are jointly measured, if role and position variables account for no interpretive or predictive variance beyond topic and sentiment, the central premise of this article fails and the lexical paradigm it criticizes stands as adequate.

Three lines of work follow. The first concerns variation in position over time, and asks whether movement in narrated agency indexes anything beyond the discursive record, which requires co-measurement with efficacy scales and with documentary evidence of institutional uptake. The second concerns comparison across processes, and asks whether citizens' positional profiles differ according to the influence their contributions carry, and whether linguistic analysis registers that difference before it appears as withdrawal. The third concerns the measurement infrastructure itself: annotated corpora of Latin American participatory text, connotation lexicons calibrated to the regional political context, and function-word category schemes validated for Spanish. None of this can be built without collaboration among computational linguists, political scientists, designers of participatory processes and the communities that take part in them.

What this article establishes is that these questions have sufficient linguistic, psychological and normative grounding to be worth asking precisely. Whether the answers vindicate the framework is a separate matter, and the article has tried to specify what it would take to find out.

## Data Availability

The original contributions presented in the study are included in the article/supplementary material, further inquiries can be directed to the corresponding author.
